# The Unpluggable in Pursuit of the Undruggable: Tackling the Dark Matter of the Cancer Therapeutics Universe

**DOI:** 10.3389/fonc.2013.00304

**Published:** 2013-12-12

**Authors:** Richard J. Epstein

**Affiliations:** ^1^Laboratory of Genome Evolution & Informatics, The Kinghorn Cancer Centre, and Clinical Informatics & Research Centre, Department of Oncology, St Vincent’s Hospital, UNSW Clinical School, Sydney, NSW, Australia

**Keywords:** tumor suppressor genes, genetic instability, apoptosis, carcinogenesis, drug development

## Abstract

The notion that targeted drugs can unplug gain-of-function tumor pathways has revitalized pharmaceutical research, but the survival benefits of this strategy have so far proven modest. A weakness of oncogene-blocking approaches is that they do not address the problem of cancer progression as selected by the recessive phenotypes of genetic instability and apoptotic resistance which in turn arise from loss-of-function – i.e., undruggable – defects of caretaker (e.g., *BRCA*, *MLH1*) or gatekeeper (e.g., *TP53*, *PTEN*) suppressor genes. Genetic instability ensures that rapid cell kill is balanced by rapid selection for apoptotic resistance and hence for metastasis, casting doubt on the assumption that cytotoxicity (“response”) remains the best way to identify survival-enhancing drugs. In the absence of gene therapy, it is proposed here that caretaker-defective (high-instability) tumors may be best treated with low-lethality drugs inducing replicative (RAS-RAF-ERK) arrest or dormancy, causing “stable disease” rather than tumorilytic remission. Gatekeeper-defective (death-resistant) tumors, on the other hand, may be best managed by combining survival (PI3K-AKT-mTOR) pathway blockade with metronomic or sequential pro-apoptotic drugs.

## Introduction

Tolstoy’s *Anna Karenina* begins, “All happy families are alike, but all unhappy families are unhappy in their own special ways.” This is a literary way of saying that there are more ways for complex systems to go wrong than to remain right, and helps to explain why cancer remains the most challenging of human diseases. It also hints at why current approaches to drug development continue to yield frustratingly marginal benefits (1).

Common cancers arise from the progressive accumulation of common genetic errors, most of which subvert the function of normal cell-regulatory genes. If a germline defect in one of these regulatory genes predisposes to familial or heritable cancers, the nomenclature “tumor suppressor gene” has often been used. In 1997 Kinzler and Vogelstein noted that most tumor suppressor genes fall into just two functional categories: “caretaker” genes that repair DNA and maintain genetic stability, or “gatekeeper” genes that regulate cell-cycle progression and apoptosis (2). This semantic dichotomy is too simple (3), of course, given that genetic instability is exacerbated by gatekeeper gene defects that permit survival of cells which would otherwise self-destruct, whereas apoptotic resistance is worsened by caretaker defects that impair sensing of potentially lethal insults by the afferent limb of the DNA damage response (4). Nonetheless, as argued below, the potential utility of this model (5, 6) – contrasting as it does with more complex but less user-friendly models of cancer biology (7) – has not yet been exploited in clinical practice or research.

The ability of a cell to engage in oncogenic oversignaling implies selection for a pre-existing suppressor gene defect, given that normal cells with intact control pathways typically succumb to cell death as a result of constitutive hyperstimulation (8). For this reason alone, cancer treatment strategies focused solely on “driver” pathway inhibition seem likely to fail – for no sooner is the proverbial plug extracted from the driver pathway than the underlying apoptotic gene defect permits selection for heterologous pathway upregulation and/or additional oncogenic events, manifesting as a rapidly proliferative (high Ki67) tumor outgrowth reflecting the suppressor gene mutation burden (9). This problem is made even worse by coexisting caretaker defects that speed selection and cell adaptation – a useful coping mechanism for germline (species) evolution (10), but yet another therapeutic hurdle for restoring phenotypic stability to growing neoplasms.

A further impediment to the vision of personalized cancer medicine is that the heterogeneity of molecular defects within tumors far exceeds the existing range of targeted drugs. Broader characterizations of dominant tumorigenic pathway dysfunction, reflecting the relative overactivity of major signaling cascades – those mediated by RAS-ERK (replication) vs. PI3K-AKT (survival) signaling – could usefully guide clinicians as to best treatment decisions; with regard to the latter pathways, for example, whether to prioritize replication arrest and thus slow progression of genetic instability, or instead to focus on apoptotic sensitization by blocking mTOR upregulation originating from, say, *PIK3CA* mutations or heregulin- and insulin-related oversignaling (11– 13). An example is detailed in our recent report of a patient with refractory progressive colorectal cancer which was mismatch-repair (MMR)-deficient, *KRAS* wild-type, and *BRAF*^V600E^-mutant, who appeared to benefit from a small-molecule BRAF inhibitor (14) only when an epidermal growth factor receptor (EGFR) inhibitor was co-prescribed to block this interfering anti-apoptotic pathway (15).

## The Clinical Challenge of Suppressor Gene Defects

The crisis of blockbuster drug development in today’s omics-obsessed pharmaceutical industry (16) originates in part from commercial strategies that rely on unplugging “addicted” oncogene targets as a seductively simple solution to the cancer problem (1, 17). This approach may work for rare oncogene-expressing tumor types with low genetic instability, such as chronic myeloid leukemia or medullary thyroid cancer (18), but common cancers are complicated by a shifting balance: for every oncogenic driver pathway, there is a permissive spectrum of suppressor gene defects lurking in the molecular background which begin to erode survival gains as soon as signaling blockade is achieved. These suppressor defects are loss-of-function in type, and hence undruggable by standard pharmacologic approaches (19) which continue, for sound technical reasons, to focus on enzyme and/or receptor inhibition (20). As indicated in Table 1, clinical use of such drugs selects rapidly for a cascade of downstream control defects that accelerate both tumor resistance and disease progression (21).

**Table 1:** **Sequence of steps in cancer drug development**.

Research phase	Therapeutic priority
Basic	Identification of tumor-specific oncogenic “driver” target
Translational	Synthesis of target-specific driver-inhibitory drug
Clinical	Empirical characterization of inhibitor-induced secondary resistance problems, reflecting increased apoptotic threshold (gatekeeper pathway defect), and/or increased genetic instability (caretaker pathway defect)

*The initial step (basic research) involves identification of a pro-mitotic “driver” protein implicated in tumor growth. The next (translational) step involves isolation of a lead compound or synthetic drug capable of inhibiting functional activity of the driver protein – usually an enzyme or receptor. The final steps involve clinical trials assessing not only the drug’s safety and dosimetry (phase 1) and the tumorilytic efficacy (phase 2), but also the durability or otherwise of tumor-inhibiting efficacy, and hence any survival gain compared to standard treatments (phase 3). Unfortunately, dynamic reductions in the durability of drug control – as distinct from *de novo* resistance – often arise from secondary selection for repair defects (accelerating tumor progression) and/or apoptotic defects (reducing tumor response)*.

The challenge of networking a drug-based solution to this Humpty-Dumpty-like panoply of covert genetic errors (22) – not least in mutation-rich tumors such as smoking-related lung cancer, or inflammation-induced hepatocellular carcinoma – has long been consigned to the “too-hard basket” by clinicians and Big Pharma alike. The most immediate prospects for progress may well lie in the field of lifestyle-related cancers (e.g., breast, colon, prostate) where the burden of hard-wired gene defects is an order of magnitude lower than in carcinogen-dependent tumor types (23). This logic is illustrated by the observation that papillomavirus-associated oropharyngeal cancers, which are specifically initiated by viral E6/E7 oncoprotein blockade of p53 and pRb gatekeeper gene function, have a superior prognosis to smoking-induced cancers of the same anatomic site and morphology (24).

So where should we go from here? The caretaker/gatekeeper model of tumor progression provides a starting point. Consider, for example, tumors caused by mutations of caretaker gene function, such as MMR-defective colorectal cancer, whether sporadic or familial (25). These tumors are associated with numerous somatic mutations (23) consistent with their defining microsatellite instability (MSI); despite this, they are associated with better prognosis than stage-matched microsatellite-stable (MSS) tumors (26), consistent with a lack of major gatekeeper defects driving metastasis (27, 28). Adjuvant fluoropyrimidine chemotherapy appears of less benefit in MSI tumors (29) – partly reflecting the more favorable natural history of these cancers, to be sure, but plausibly also reflecting failure of misincorporated antimetabolites to trigger MMR and hence activate programed cell death (4) – whereas retrospective analyses have suggested that microenvironment-modulating drugs could selectively improve survival in this tumor subtype (30).

Conversely, germline mutations of the gatekeeper gene *TP53* give rise *in vivo* to breast cancers which are *HER2*-overexpressing in ∼80% cases (31), supporting the view that apoptotic defects are a prerequisite for clonal outgrowth of such tumors (8), while also suggesting a clinical opportunity to reduce this defect and thus enhance chemosensitivity. Since the HER2 protein heterodimerizes preferentially with HER3 (32) – which, by virtue of numerous Y*XX*M peptide motifs in its carboxyterminal tail (33), is a potent driver of the anti-apoptotic PI3K-AKT-mTOR pathway (34) – therapeutic inhibition of HER2-initiated signaling can be predicted to augment tumor cell kill by chemotherapy. This accords with experience in the clinic, where trastuzumab (Herceptin™) greatly increases chemotherapy efficacy (35) yet confers only minor clinical benefits when used as monotherapy (36, 37). Moreover, trastuzumab resistance is acquired during treatment via new activating *PIK3CA* mutations and/or *PTEN* losses (38–40), with drug sensitivity capable of being restored by downstream blockade of this pathway (41). This example illustrates how “undruggable” apoptotic defects (such as those mediated by mutant *TP53*) may be remedied by targeting more readily druggable heterologous pathway upregulation (such as mTOR signaling).

This approach could be extended to less well-defined clinical contexts by elucidating broad patterns of oncogenic pathway activation using phosphoproteomic fingerprinting (42). Genome sequencing analyses of lifestyle cancers have confirmed that the usual genetic stigmata of tumorigenic transformation comprise a small group of aberrations (43–46), consistent with the model of tumor suppressor gene loss proposed above: namely, anti-apoptotic dysfunctions affecting either *TP53* (including those secondary to *BRCA* mutations (47) or *PTEN*; gain-of-function mutations affecting *KRAS* or *PIK3CA*; or MSI (flagged by low MMR expression on histochemistry) with or without activating *BRAF* mutations (48). If tomorrow’s clinicians can interpret this unambiguous molecular language, rational treatments may indeed become customizable for patients.

Further support for the notion of suppressor-led therapeutics comes from studies showing restoration of hormone-sensitivity to breast (49) and prostate cancers (50) using mTOR pathway inhibition. *PTEN* deletions are amongst the commonest of all genetic lesions in hormone-dependent cancers (51), and activation of *PIK3CA*-inducible biomarkers correlates with both preclinical (52) and clinical response to the oral mTOR inhibitor everolimus (53). The logic of using a downstream inhibitor to block the consequences of an otherwise undruggable upstream gene defect is thus supported, reinforcing lessons learned from constitutive *KRAS*-mutant-activated colorectal cancer in which upstream RAS-ERK blockade by EGFR antibodies is ineffective (54). Different lesions upregulating the mTOR signaling pathway may have non-identical therapeutic implications (55), however, emphasizing that prediction of effective tumor targeting may come to involve more than a one-off genetic predictive assay.

## Discovery of a “BRCA Attacker”?

Recent advances in the field of *BRCA* mutant cancers have likewise kindled fresh interest in the nascent field of suppressor-based therapeutics (56). Like MMR gene mutations, *BRCA* mutations are relatively common caretaker defects in the population at large, with this heterozygote frequency perhaps having been maintained by lethal epidemics such as bubonic plague (57). This raises the counterintuitive hypothesis that *BRCA* mutations may give rise to a population survival advantage under extreme environmental selection pressures – a hypothesis supported by an analysis of spontaneous abortions that showed an unexpected reduction in lifetime miscarriage frequency among *BRCA* mutant carriers (25.2%) compared to non-carriers (29.1%), correlating with a higher number of full-term pregnancies (2.15 vs. 1.94) (58). This is also consistent with our model of programed genetic instability, which posits that an evolutionarily conserved sequence-dependent (CpG-based) predisposition to germline caretaker gene mutation permits genomic plasticity and species adaptivity – i.e., positive selection facilitated by a mutator phenotype (59) – in response to changes in environmental stress (10). The mutability of caretaker genes such as *BRCA1/2* may thus be a two-edged sword depending on the genomic context, with survival gains for an adapting species under apoptotic stress (47) ultimately overriding the minor mortality costs of cancer in older individuals due to somatic genetic instability.

Viewed from this evolutionary perspective, the discovery that therapeutic inhibition of poly(ADP-ribosyltransferase) polymerase (PARP) enzymes selectively enhances cytotoxicity in *BRCA* mutant tumor cells deficient in homologous recombination (60–62) – the novel paradigm of synthetic lethality (63) – merits cautious appraisal (64, 65). Like BRCA proteins (66), PARPs are implicated in the maintenance of genome stability (67), either by forming a “sugar plug” across DNA single-strand breaks when enzymatically cleaved during a potentially cell-lethal damage response, or else by blocking replication and thus enhancing repair when remaining bound to DNA while still intact. The problem of instability-induced resistance therefore remains pivotal to the clinical promise (i.e., the survival benefit – as distinct from tumor response) of PARP inhibitory therapy for *BRCA* mutant disease (68). Since both BRCA1/2 and PARP1/2 proteins are required for normal genetic stability, reduced (defective or inhibited) BRCA (69) and/or PARP function could plausibly accelerate resistance (70) and/or disease progression (68), thereby offsetting short term benefits of PARP1/2 blockade in certain subsets of *BRCA* mutant tumors (71).

Chromosomal instability (CIN) markers such as telomere allelic imbalance or quadriradial chromosomes indicate the “BRCAness” (i.e., genomic instability due to impaired error-free homologous recombination/repair) of tumors – even tumors lacking *BRCA* gene mutations, such as those with *BRCA* gene promoter methylation (72). Of note, such BRCAness correlates positively with tumor response rates to alkylating chemotherapy drugs like cisplatin – as indeed does *PARP1/2* gene knockout in mice – yet negatively with responses to taxane-based chemotherapies (73). Similarly, the clinical observation that *BRCA*-mutant patients do not exhibit hypersensitivity to ionizing radiation (74) raises a more nuanced interpretation of this genotype than that of a damage sensitization predictor. Moreover, given that BRCAness implies a default (salvage) increase in error-prone DNA repair, such as must presumably be associated with greater genetic instability, high chemotherapy response rates of such tumors (75) may not necessarily yield overall survival benefit. Notwithstanding these caveats, the potential contribution of combined PARP inhibitor and alkylator chemotherapy will remain an important priority for clarification in the palliative context.

## Putting Humpty Dumpty Back Together

The insights outlined above point to the emergence of an exciting new era in cancer management. For the foreseeable future, however, progress against common solid tumors is likely to remain incremental rather than transformational for the following reasons.

First, there will remain serious difficulties in rectifying complex loss-of-function molecular defects on a durable basis, especially in high-grade poorly differentiated carcinomas with heavy mutation loads affecting both caretakers and gatekeepers.

A second and related problem concerns the “moving goalposts” of the cancer problem, reflecting therapeutic frustration over time due to the target-evading double-hit of genetic instability and apoptotic resistance. Modern genomic fingerprinting assays provide an impressive molecular snapshot of malignant processes at any one time, but development of a more dynamic “molecular film” technology is now needed to predict disease biology as it affects treated patients in real time. An ideal management strategy will be to minimize tumor genomic instability by slowing cell replication to the point of dormancy – i.e., as distinct from current ablative strategies of killing the most apoptosis-sensitive tumor cells upfront, inadvertently driving Gompertzian growth and metastasis of the remaining resistant cells in the longer term (76). This strategy would seem most plausible in lifestyle-related cancers, which appear driven in part by environmentally sensitive epigenetic defects (77). Changing clinical trial strategies to focus less on response and more on disease stabilization is a key step in this direction, whereas false economies in pursuing commercially optimistic but biologically misguided designs need conscious avoidance.

Third, it is vital to be aware that cancer growth is regulated by numerous factors outside the tumor itself – e.g., metabolic, endocrine, immune, stromal – which cannot be analyzed or predicted by even the most comprehensive tumor-centric analyses. Cancer is not like an infection which can be cultured to determine its drug sensitivity *in vitro*; rather, it is a disease of a multicellular biological system in which breakdown of regulatory crosstalk between normal and transformed cells is the primary problem (78). Immune modulation may provide one such extra-tumoral approach to “immunogenic” malignancies such as renal cell carcinoma or melanoma (79), though this strategy could prove less relevant to lifestyle-related or smoking-related neoplasms. Deepening insights into the independent tumorilytic sequelae of dieting (reduced insulin axis survival signaling), fat reduction (reduced pro-inflammatory NFκB signaling), and exercise (increased pro-apoptotic AMPK pathway activation) may help lead the way toward this objective (80).

Similarly, the judicious adjuvant use of cytotoxic chemotherapy to induce stromal toxicity and thus trigger micrometastatic apoptosis due to paracrine loop disruption – for example, in the relatively unexplored context of high-grade prostate cancer – remains as rationally justified as more costly molecularly targeted initiatives. Indeed, it is becoming understood that toxicity can often be a reliable predictor of anticancer drug benefit (81, 82), consistent with a role for normal cell interaction in tumor viability and progression. Other drug classes that may not affect tumor response or survival in the metastatic setting – e.g., protease inhibitors, or antagonists of G-protein-coupled receptors (83) – could still provide first-in-class targets by which to block metastasis in the adjuvant setting, thus improving survival.

The implications of this paradigm shift for clinical practice could prove to be profound. If a given tumor, such as glioblastoma, is characterized by high apoptotic resistance but low genetic instability, treatment may be focused on lowering the apoptotic threshold, e.g., by using PI3K-AKT-mTOR inhibitors (84). On the other hand, if a tumor exhibits high genetic instability driving widespread metastasis – e.g., *BRAF* mutant melanoma – then reducing tumor replication by damping down RAS-ERK signaling could slow worsening instability (85). Additional examples illustrating this clinical research strategy are shown in Table 2.

**Table 2:** **Examples of tumor types differing in extent of caretaker/gatekeeper suppressor gene dysfunction, together with suggested therapeutic strategies**.

High-instability tumors		Apoptosis-resistant tumors		“Double-trouble” tumors	
Examples	Predicted treatment strategy	Examples	Predicted treatment strategy	Examples	Predicted treatment strategy
Premenopausal ER-positive, PR-negative, *BRCA* mutant, moderate Ki67, invasive ductal breast cancer (luminal B)	Adjuvant: bolus CT (to disrupt stromal-epithelial micro-metastatic niches), then continuous HT (≥5 years)	Postmenopausal ER/PR-rich, *BRCA* wild-type, low-Ki67, *PTEN*-deleted or *PIK3CA*-mutant, invasive ductal breast cancer (luminal A)	Adjuvant: long-term continuous HT (≥10 years)	Triple-negative (ER-absent) invasive ductal breast cancer: *BRCA* mutant, *TP53* mutant, high Ki67 (basaloid)	Adjuvant: bolus CT (to disrupt stromal-epithelial micro-metastatic niches)
			Palliative: sequential HTs, plus mTORi on progression	
	Palliative: sequential HTs, then sequential alkylator-based CTs, plus PARPi on progression	*HER2*-amplified, ER-poor, *TP53* mutant, moderate Ki67, invasive ductal breast cancer	Adjuvant: HER2i-primed bolus CT	
			Palliative: HER2i-primed metronomic CT, plus mTORi on progression		Palliative: sequential CTs using alkylator-based regimens, plus PARPi, or mTORi on progression
		ER-rich, *BRCA* wild-type, *PIK3CA*-mutant, low-Ki67, *CDH1*-mutant, classic lobular breast cancer	Adjuvant: continuous HT (≥5 years)	
			Palliative: sequential HTs, plus mTORi on progression	
Proximal colorectal cancer, MSI, *TP53* wild-type, *BRAF* mutant, *KRAS* wild-type	Adjuvant: bolus alkylator-based CT	Distal colorectal cancer, MSS/CIN, *TP53* mutant, *BRAF* wild-type, *KRAS* mutant	Adjuvant: bolus fluoropyrimidine + alkylator-based CT	MSI + CIN colorectal cancer	Adjuvant: bolus fluoropyrimidine + alkylator-based CT
	Palliative: sequential CTs using antibodies to VEGF or EGFR; then dual BRAF-EGFR blockade		Palliative: sequential CTs using antibodies to VEGF as needed		Palliative: sequential CTs using sensitizing antibodies to VEGF continuously

*CIN, chromosomal instability; CT, chemotherapy; EGFR, epidermal growth factor receptor; ER, estrogen receptor; HER2I, HER2 inhibitor; HT, hormonal therapy; MSI, microsatellite instability; MSS, microsatellite stability; mTORi, mTOR inhibitor; PARPi, PARP inhibitor; PR, progesterone receptor; VEGF, vasoactive endothelial growth factor*.

## Conclusion

There are now growing justifications for incorporating assessments of genetic instability and apoptotic resistance into therapeutic anticancer strategies and clinical trial designs. It is important to concede that not all tumors may lend themselves to this relatively simple classification, and more sophisticated modeling paradigms will undoubtedly be developed in the future. Even in the short term, however, greater awareness of these important phenotypic variables may improve the prospects for drug-induced disease control and survival gain in a significant subset of cancer patients.

## Conflict of Interest Statement

The author declares that the research was conducted in the absence of any commercial or financial relationships that could be construed as a potential conflict of interest.

## Abbreviations

CIN, chromosomal instability; CT, chemotherapy; EGFR, epidermal growth factor receptor; ER, estrogen receptor; HT, hormonal therapy; MMR, mismatch repair; MSI, microsatellite instability; MSS, microsatellite stability; PARP, poly(ADP-ribosyltransferase) polymerase; PR, progesterone receptor; VEGF, vasoactive endothelial growth factor.
